# Deep Neural Regression Prediction of Motor Imagery Skills Using EEG Functional Connectivity Indicators

**DOI:** 10.3390/s21061932

**Published:** 2021-03-10

**Authors:** Julian Caicedo-Acosta, German A. Castaño, Carlos Acosta-Medina, Andres Alvarez-Meza, German Castellanos-Dominguez

**Affiliations:** 1 Signal Processing and Recognition Group, Universidad Nacional de Colombia, Manizales 170001, Colombia; cdacostam@unal.edu.co (C.A.-M.); amalvarezme@unal.edu.co (A.A.-M.); cgcastellanosd@unal.edu.co (G.C.-D.); 2 Grupo de investigación Cultura de la Calidad en la Educación, Universidad Nacional de Colombia, Manizales 170001, Colombia; gacastanod@unal.edu.co

**Keywords:** motor imagery, BCI inefficiency, functional connectivity, neural regression, media and information literacy

## Abstract

Motor imaging (MI) induces recovery and neuroplasticity in neurophysical regulation. However, a non-negligible portion of users presents insufficient coordination skills of sensorimotor cortex control. Assessments of the relationship between wakefulness and tasks states are conducted to foster neurophysiological and mechanistic interpretation in MI-related applications. Thus, to understand the organization of information processing, measures of functional connectivity are used. Also, models of neural network regression prediction are becoming popular, These intend to reduce the need for extracting features manually. However, predicting MI practicing’s neurophysiological inefficiency raises several problems, like enhancing network regression performance because of the overfitting risk. Here, to increase the prediction performance, we develop a deep network regression model that includes three procedures: leave-one-out cross-validation combined with Monte Carlo dropout layers, subject clustering of MI inefficiency, and transfer learning between neighboring runs. Validation is performed using functional connectivity predictors extracted from two electroencephalographic databases acquired in conditions close to real MI applications (150 users), resulting in a high prediction of pretraining desynchronization and initial training synchronization with adequate physiological interpretability.

## 1. Introduction

Motor imaging (MI) is the dynamic cognitive capability of generating mental movements without executing them. This mental process triggers the neurocognitive mechanisms that underlie voluntary movement planning, similar to how the action is performed realistically. MI has been proposed as a reliable tool in acquiring new motor skills to increase sports performance and physical therapy [1,2,3,4], in the development of professional motor skills learning [5], and in improving balance and mobility outcomes in older adults and children with developmental coordination disorders [6,7], among others. There is sufficient experimental evidence that MI induces recovery and neuroplasticity in neurophysical regulation as the basis of motor learning [8] and educational fields [9]. Concerning this aspect, the Media and Information Literacy approach has been proposed by the United Nations Educational, Scientific, and Cultural Organization (UNESCO) to gather several vital human development capabilities. In practice, MI tasks are commonly solved from electroencephalography (EEG) records, which provide noninvasive measures and flexible portability at a relatively low-cost. However, EEG signals lack a suitable spatial resolution, not to mention the inter and intra-subject variability regarding the somatosensory cortex’s responses. Specifically, there is no consistency in the patterns among different subjects. Indeed, the variability arises within a session for the same subject because of a non-stationary, nonlinear, and low signal-to-noise ratio of EEG signals [10]. Together with frequently used small sample datasets, all of these factors reduce MI systems’ performance based on EEG [11,12].

An enhanced approach to addressing this EEG data complexity is to conduct multiple training sessions to refine the modulation of sensorimotor rhythms (SMR). Nonetheless, the inter-subject variability, together with uncertain long-term effects and the apparent failure of some individuals to achieve self-regulation, makes a non-negligible portion of users (between 15% to 30%) develop insufficient coordination skills even after long training sessions. This inadequate performance of most brain-computer interface (BCI) systems (*BCI inefficiency*) poses a challenge in MI research [13]. To address this problem, the BCI performance model is enhanced in two directions: (*i*) Developing guidelines in neural testing set-ups, practice, and instructions to ensure better performance of brain responses; and (*ii*) Promoting evaluation tools to forecast the system performance may help identify the core issue of variability to incorporate compensating actions for the inefficiency when solving BCI-based tasks. In particular, a calibration strategy can be added, working hand in hand within the training stage. Therefore, it is possible to adapt the decoding scheme with an explicit brain pattern [14], highlighting relevant BCI predictors to decrease training efforts and encourage user-centered MI [15]. To date, several electrophysiological indicators have been reported to anticipate the MI inefficiency, like the direct assessment of the SMR, which extracts the power spectral density (PSD) from the resting wakefulness at motor cortex locations [16]; a measure of the PSD uniformity of the resting-state data using spectral entropy [17,18]; and the PSD-based estimate to assess the dis/similarity (connectivity) of EEG signals at different locations in an attempt to understand the interdependency between functional and structural networks of corresponding cortical brain structures (like spectral coherence [19,20] or coherence-based correntropy spectral density [21]), among others. To tackle the influence of artifacts and intertrial/inter-subject amplitude variability, phase-based relationships (phase synchronization) are more desirable as a functional connectivity (FC) measure of spatially distributed regions, dynamically interacting in accomplishing a mental task [22]. It has been proved that the functional connectivity features measured by the phase lag index (or its weighted version—*wPLI* and phase-locking value *PLV*) can discriminate between different MI tasks [23,24].

Therefore, predicting motor performance from the resting motor-system functional connectivity can be determined as in [16], showing that the efficient brain reconfiguration corresponds to a better MI performance [25,26]. Nevertheless, several conditions can affect their correct estimation and introduce spurious contributions, giving a potentially distorted measure of the real interactions (termed spurious connectivity) [27]. Thus, FC estimation is highly time-dependent and fluctuates within multiple timescales, yielding inter-subject variations that remain a substantial problem [28]. Specifically, the obstacles related to volume conduction and noise perturbations cause phase synchronization to incorporate thresholds applied to these FC measures to improve the connection sets’ discriminative ability. However, the threshold selection is generally far from being an automated procedure for big datasets [29]. Undeterred by the promising evidence, there is a need to understand the learning mechanisms and the brain network reorganization, aiming to support the efficiency of BCI systems [30].

As regards the prediction model, several regression methods are available for prognosticating MI accuracy from neurophysiological variables like simple and multiple linear regression [31,32], stepwise regression [33], kernel regression [34], and (kernel) support vector machine regression [35], among others. Additionally, there is increasing use of regression approaches with neural networks that can be applied to the raw EEG data, simplifying BCI’s design pipelines by removing the need to extract features manually. However, several aspects degrade the prediction model performance, such as the fact that FC measures are prone to be influenced by outliers, which are to be removed before calculating correlations [36]. Another drawback is the inter-trial variability of MI data (with a notable increase in subjects having low MI skills), which restricts prediction models with single-trial EEG data [37]. One more issue influencing the regression model is the user’s categorization depending on their SMR activity (predictor) and classifier performance (target response) during the MI runs. Users are frequently adjusted to two partitions (skilled and non-skilled) divided by a single target value given in advance, as in [38]. Still, as the number of subjects tested increases, the range of FC changes also rises. The partition-based method should also be sensitive in detecting predictor differences among subject clusters [39,40]. Therefore, the need for clustering into more partitions becomes more evident, as shown in [41]. Lastly, the correlation coefficient (reflected in *r*-squared) is often applied to assess the prediction shape, while the *p*-value levels its statistical significance that can be implemented through several test procedures, as developed in [42]. A common issue in neural network regression models, trained with small samples in MI studies, is their fitting to spurious residual variation (overfitting), apart from a controversial interpretation of *p*-values [43].

Here, to increase the prediction performance of the baseline linear regression models, we develop a deep network regression model devoted to prognosticating Motor Imagery Skills using EEG Functional Connectivity Indicators, appraising three procedures: leave-one-out cross-validation combined with Monte Carlo dropout layers, subject clustering of MI inefficiency, and transfer learning connecting neighboring runs. Our approach comprises functional connectivity predictors extracted from electroencephalographic signals to favor the data interpretability. To deal with the risk of overfitting prediction assessments because of the deep learning framework, we intend to preserve as much information as possible from the measured scalp potentials. Thus, to reach competitive values of prediction errors achieved by the leave-one-out cross-validation scheme, we introduce the following procedures: (i) Monte Carlo dropout layers to decrease the probability that the learned rules from specific training data cannot be generalized to new observations; (ii) Subject efficiency clustering to adapt the DNR estimator more effectively to complex EEG measurements inherent to BCI inefficiency subjects; (iii) For Prediction of Initial-training Synchronization, transfer learning of the weights inferred at the predecessor run to deal with the few-trial sets. The validation is performed in two MI databases (150 users) acquired in conditions close to real MI applications. Obtained results show how our approach can achieve a high prediction of pretraining desynchronization and initial training synchronization with adequate physiological interpretability. We further compared the DRN predictor prediction performance (on average, 0.8) with the results obtained by linear regression models that are reported, at least for DBI, in the baseline work [44], presenting values of R-squared not exceeding 0.54.

The rest of the paper is organized as follows: Section 2 briefly discusses the regression prediction model’s theoretical background. Section 3 describes the experimental set-up, including both datasets evaluated. Section 4 presents the assessment of Deep Regression Network performance and discusses the findings obtained to predict pretraining desynchronization and initial training synchronization. Lastly, Section 5 concludes the paper.

## 2. Methods

### 2.1. Electrophysiological Predictors Based on Phase Synchronization Relationships

Initially, we consider predictors based on the following two widely-used FC measures of phase synchronization between every pair of EEG channels $x_{c},x_{c^{\prime }}\;\in \;R^{T}$ ($\forall c,c^{\prime }\;\in \;C,c\;\neq \;c^{\prime }$, where C∈N is the number of channels):

**Phase Locking Value (*PLV*):** This phase coherence measure assesses the pairwise similarity relation based on states’ recurrence density, occurring between electrodes. For the single-trial analysis, *PLV* is computed by the following average over a time window [45]:

(1) $$\nu_{1}\left(c,c^{\prime }\right)=\left|E\left\{\exp(-j\left(\Delta \Phi \left(c,c^{\prime };n,t,f\right)\right)):\forall t\in T\right\}\right|,\;\nu_{1}\left(,\right)\in \left[0,1\right],$$

where $\Delta \Phi \left(c,c^{\prime };n,t,f\right)\in \left[-\pi ,\pi \right]$ is the instantaneous phase difference computed, at time instant *t* for the *n*-th trial (∀n∈N, where N∈N is the number of EEG trials), and notations $E\left\{\cdot \right\}$ and |·| stand for expectation operator and magnitude, respectively. Of note, to preserve physically meaningful, the phase signal must highlight only a given frequency oscillation $f\;\in \;R^{+}$. Here, this is achieved utilizing the convolution with a narrow band complex Morlet wavelet through the continuous wavelet-transform [46].

**Weighted Phase Locking Index (*wPLI*):** This indicator quantifies the pairwise phase difference distribution’s asymmetry by averaging across the trial set. Then, the *wPLI* is computed by averaging over the trial ensemble, as follows:

(2) $$\nu_{2}\left(c,c^{\prime }\right)=\frac{|E\left\{ℑ\{S\left(c,c^{\prime };n,f\right)\}:\forall n\;\in \;N\right\}|}{E\left\{|ℑ\{S\left(c,c^{\prime };n,f\right)\}|:\forall n\;\in \;N\right\}},\;\nu_{2}\left(,\right)\in \left[0,1\right]$$

where $S(c,c^{\prime };n,f)\;\in \;C$ is the cross-spectral density based on Morlet wavelets and ℑ{·} stands for the imaginary part of a complex-valued function. *wPLI* is assumed to deal with the presence of volume-conduction, noise, and sample-size bias [47].

### 2.2. Construction of Brain Graph Predictors

We also consider the predictors that involve a generic approach to characterizing brain activity using undirected graph theory. These predictors describe complex systems’ properties by quantifying their respective network representations’ topologies. In large-scale brain networks, the node-set (noted as ${\left\{\varphi_{r}\left(c\right)\;\in \;R\right\}}_{r\;\in \;Z}$) usually designates brain regions holding V=C(C−1)/2 paired (undirected) links. The following weighted network indexes are extracted from the phase synchronization-based relationships (spatiotemporal dependences) [48]:

– **Strength** is a local-scale property that accounts for the number of links connected to each node, computed as follows:
  (3) $$\varphi_{1}\left(c\right)=CE\left\{\nu \left(c,c^{\prime }\right):\forall c^{\prime }\in C,c^{\prime }\neq c\right\}.$$
– **Clustering Coefficient** is a global-scale property that indicates the tendency of a network to form tightly connected neighborhoods, measuring the segregation brain’s ability for specialized processing within densely interconnected regions, computed as follows:
  (4) $$\varphi_{2}\left(c\right)=\frac{1}{C^{2}}E\left\{\frac{\pi_{c}}{E\left\{\hat{\nu }\left(c,c^{\prime }\right)E\left\{\hat{\nu }\left(c,c^{\prime }\right)-1\right\}\right\}}:\forall c\in C,c^{\prime }\neq c\right\}$$
  where the binarized connection value (connection status) $\hat{\nu }\left(c,c^{\prime }\right)\;=\;1$, if $\nu (c,c^{\prime })>0$, otherwise, $\hat{\nu }\left(c,c^{\prime }\right)\;=\;0$. $\pi_{c}\;\in \;N$ is the number of triangles neighboring the *c*-th node.

### 2.3. Regression Network Models

Let X∈X (termed *predictor*) and Y∈Y (*response*) be a couple of random variables for which the mutual dependence $\tilde{y}\in Y=\xi \left(x\in X\right)$ is assessed through the approximating function (termed *regressor*) ξ:X→Y. Namely, let $\left\{x_{m}\;\in \;X,y_{m}\;\in \;Y:m\;\in \;M\right\}$ be the corresponding composite observation set, across M∈N subjects, the following optimization framework allows fixing the regressor as:

(5) $$w^{*}=\min_{w}E\left\{∥y_{m}-\xi \left(x_{m};w\right){∥}_{2}:\forall m\in M\right\},$$

where w is an unknown parameter vector fitting the data most closely in terms of the $\ell_{2}$-norm.

For implementing the data-driven estimator in Equation (5), we employ the *Deep Regression Network* (DRN) developed in [49] that jointly extracts and performs the regression analysis, as follows:

(6) $$\begin{array}{cc} \; & \min_{w}E\left\{∥(y_{m}-\left(\xi_{J}\circ \xi_{J-1}\circ \cdots \circ \xi_{1}\right)\left(x_{m}\left(f\right);w\right){∥}_{2}^{2}:\forall m\;\in \;M\right\}, \end{array}$$

where $\xi_{j}$ is the *j*-th layer (j∈J) and ∘ stands for function composition. Notation $x_{m}\left(f\right)$ describes the connectivity predictors extracted in each frequency rhythm *f* while $y_{m}$ contains the accuracy response of *m*-th subject.

## 3. Experimental Set-Up

The methodology for enhanced prediction of motor imagery skills using functional connectivity indicators is evaluated under a regression model to predict the bi-class accuracy response of subjects, embracing the following stages: (*i*) Predicting capability estimation of the pre-training desynchronization under a conventional linear regression model, testing different scenarios of input arrangements to improve the system performance; (*ii*) Prediction assessment of the pre-training desynchronization under the data-driven network regression model; (*iii*) Enhanced network prediction assessment using leave-one-out cross-validation combined with Monte Carlo dropout layers and clustering of subject inefficiency; (*iv*) Enhanced network regression prediction of initial-training synchronization with an additional transfer learning procedure.

The pre-training desynchronization assesses the relationship between the bi-class accuracy response and the electrophysiological indicators extracted from resting wakefulness data. We employ either resting-state or task-negative state before the cue-onset of the conventional MI trial timing for evaluation purposes. Besides, as the target response, we compute each subject’s classifier accuracy in distinguishing either MI class using the short-time sliding feature set extracted by the Common Spatial Patterns (CSP), which maximizes the class variance. To accurately extract the subject EEG dynamics over time, the sliding window is adjusted to 2 s, having an overlap of 50%.

On the other hand, the pre-training desynchronization predictor relies on the fact that the change in neural activity, intentionally evoked by a mental imagery task, shows certain regularities through training runs or sessions. Accordingly, the pre-training indicator of neural desynchronization attempts to anticipate the MI responses evoked within every run’s wakefulness data.

### 3.1. MI Databases Description and Preprocessing

**Figure 1:**
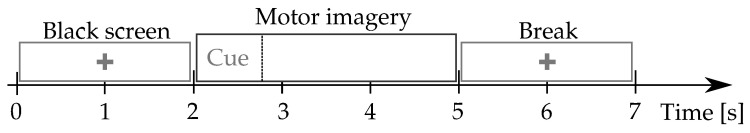
Dolutegravir may inhibit HIV-resistant viruses from becoming archived within viral reservoir.

**Giga-DBI:** This MI dataset is publicly available at (http://gigadb.org/dataset/100295, accessde on 30 January 2021). It gathers EEG records from fifty subjects (M=50), fixing the well-known 10-10 electrode configuration with C=64 channels. The signal x(c) comprises T=7 s, at $F_{s}\;=\;512$ Hz sample frequency. The MI protocol (see Figure 1) starts with a fixation cross shown on a black screen for 2 s. Further, a cue instruction is displayed depending on the MI instruction (label), which appears randomly within 3 s. For concrete testing, the cue asked to imagine moving his fingers, starting from the index finger and reaching the little one. Afterward, a blank screen is visible at the beginning of a break period (shown randomly between 6.1 and 6.8 s). Each MI run composes over 20 trials and a written cognitive quiz [50]. Every subject performed five runs (on average) and a single-trial resting-state recording, lasting 60 s.

**Figure 2:**
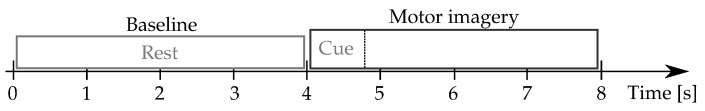
Dolutegravir may inhibit HIV-resistant viruses from becoming archived within the viral reservoir.

**Physionet-DBII:** This database, publicly available at (https://physionet.org/content/eegmmidb/1.0.0/, accessde on 30 January 2021), holds M=105 volunteers who properly performed the left and right-hand MI tasks, collecting a total average of 46.62±0.96 trials per subject. Besides, two one-minute baseline records are captured concerning a resting state trial (with eyes open and closed, respectively). The 64-channel EEG signals were recorded using the 10-10 international system, and sampled at $F_{s}\;=\;160$ Hz. Figure 2 describes the motor imagery timing.

Every raw EEG channel of either database was band-pass filtered in the frequency range f∈ [4–40] Hz, covering the sensorimotor rhythms considered (θ,μ,β). Then, the band-passed EEG data are spatially filtered by a Laplacian filter centered on the selected electrode to improve the spatial resolution of EEG recordings, avoiding the influence of noise coming from neighboring channels and thus addressing the volume conduction problem (This filtering procedure was carried out using *Biosig Toolbox* that is free available at http://biosig.sourceforge.net, accessde on 30 January 2021). Further, the electrophysiological indicator set, $\left\{\varphi_{r}\right\}$, based on phase synchronization is extracted using the *MNE package* in Python, while the graph predictors are estimated using the Brain Connectivity Toolbox (brain-connectivity-toolbox.net).

### 3.2. Deep Network Regressor Set-Up and Performance Evaluation

**Figure 3:**
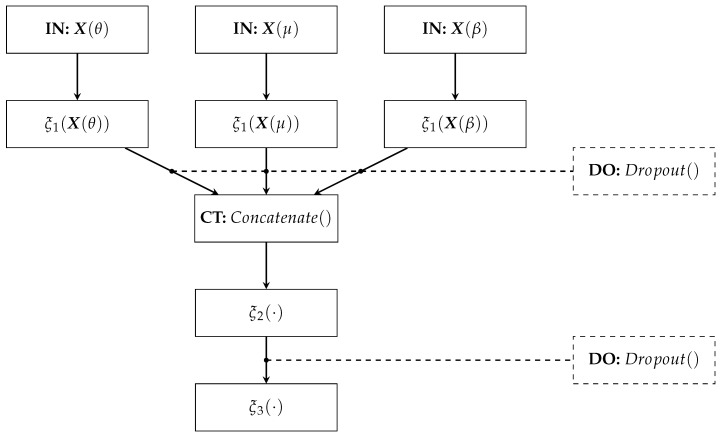
Deep Network Regressor’s architecture.

The proposed Deep Regression Network architecture comprises (see Figure 3):

– **IN**: We consider two inputs-layer arrangements: multivariate indicator $X(f)\;\in \;R^{C\times M};f\in F$ (Being *C* the electrode and links number when using graph indexes and FC, respectively).
– $\xi_{1}$: The first dense layer codes the input relevant patterns from phase synchronization features. Here, we fix the number of neurons as h=⌜1.5C⌝ neurons, where ⌜·⌝ stands for the ceiling operator. A *tanh*-based activation is employed to reveal non-linear relationships.
– **CT**: A concatenate layer is applied to append the resulting feature maps from the set of patterns extracted in $\xi_{1}$. In particular, all phase synchronization-based features (coded as connectivity matrices) are stacked into a single block, sizing hF.
– $\xi_{2}$: This fully-connected layer aims to preserve the predicted patterns assembled in the CT layer to fed a linear regressor. The number of neurons is fixed as ⌜0.5hF⌝. Again, the *tanh* is used as activation function.
– **$\xi_{3}$**: A one-neuron layer with linear activation is used to predict the MI skill value ${\tilde{y}}_{m}\in R$.
– **DO:** This Dropout layer randomly skips neurons according to drop rate. We fix the drop rate at 0.2 empirically.

For measuring the relationship between the response variable and the composite predictor, we build the set ${\left\{y_{m}^{*},y_{m}\right\}}_{m=1}^{M}$, where ${\tilde{y}}_{m}^{*}\in R$ is computed using our Deep Learning Regressor following a leave-one-out cross-validation strategy along with the *M* subjects. The quantity measures account for the influence to predict the acceptance rate on the electrodes performed by individuals, namely, for computation of value ${\tilde{y}}_{m}^{*}$, one individual is picked out as the training set and the remaining ones as the testing set. Then, the coefficient of determination (noted as *R*${}^{2}\;\in \;R\left[0,1\right]$) is computed. Besides, a *p*-value is computed from a two-sided t-test whose null hypothesis is that the regression slope is zero [44]. It is worth noting that such a hypothesis testing is used, as in state-of-the-art works [38,44], because our Deep Learning Regressor aims to code the no consistency in the brain patterns among different subjects to favor a linear dependency between $y_{m}$ and ${\tilde{y}}_{m}^{*}$. Moreover, to provide a comparison with Neural Network-based regression strategies, the real-valued measures of *Mean Absolute Error* (MAE), and *Root Squared Error* (RMSE) are also assessed, as carried out in [51,52]:

(7) $$\begin{array}{cc} R^{2} & =1-\frac{\mathrm{var}\{y_{m}-{\tilde{y}}_{m}^{*}:\forall m\;\in \;M\}}{\mathrm{var}\{y_{m}:\forall m\;\in \;M\}} \end{array}$$

(8) $$\begin{array}{cc} \mathrm{MAE} & =E\left\{∥y_{m}-{\tilde{y}}_{m}^{*}{∥}_{1}:\forall m\;\in \;M\right\} \end{array}$$

(9) $$\begin{array}{cc} \mathrm{RMSE} & =E\left\{∥y_{m}-{\tilde{y}}_{m}^{*}{∥}_{2}:\forall m\;\in \;M\right\} \end{array}$$

where var{·} stands for the variance operator.

## 4. Results and Discussion

### 4.1. Baseline Linear Regression of Pre-Training Desynchronization

Here, we consider two scenarios of input predictor arrangements: (*i*) Matrix indicator, when computing one individual network vector to reflect the electrode contribution (termed *multichannel*); (*ii*) Vector indicator, holding a single scalar value of FC accomplished by each subject. For comparison purposes with similar reported works, we analyze two indicator approaches extracted from each individual: (*a*) *Average* that obtains the mean value over the electrode set, and (*b*) the channel with the best *R*-squared (*best channel*).

Intending to evaluate the linear regression model, Table 1 displays the values of *R*-squared and its significance (namely, *p*-value), which are calculated using the Sklearn package in Python. As seen, both indicator approaches, multichannel and average, perform below the procedure for best channel extraction from the resting-state data regardless of the brain graph predictor employed. Thus, selecting the best channel allows achieving higher values of *R*-squared with lower *p*-values within the considered frequency rhythms. In the case of the predictors directly extracted from FC measures, *PLV* and *WPLI*, the upper triangular matrix is vectorized to feed the regression, yielding a performance similar to the graph indexes. Note that the best channel approach is not reported for *PLV* and *WPLI* because of difficulties in their implementation. Consequently, the above prediction results show that DBI achieves a poor performance in predicting the pre-training desynchronization, at least, using the baseline linear regression. Besides, the DBII collection gives a much worse prediction than DBI since the former EEG data contain fewer trials (100 vs. 22 per label), more subjects (105 vs. 50), and was acquired with a much lower sampling frequency (512 vs. 150 Hz). Still, the importance of considering EEG collections with elevated complexity remains an actual problem since they are more close to the requirements of real MI applications.

We also consider the case of multiple linear regression, involving both graph index predictors. Table 2 shows the effect of multiple regression remains still controversial for the tested EEG data because no suitable values of *R*-squared can be accomplished using either scenario of input arrangement representation.

### 4.2. Network Regression Prediction of Pre-training Desynchronization

Next, we employ the multichannel matrix indicator extracted from the whole subject set to predict the pre-training desynchronization. We also analyze the joint characterization of both graph indexes (strength and clustering coefficient), concatenating their vector representations of each subject into a single supervector. Besides, we take advantage of the wide path to feed the *Wide&Deep* neural network with different training sets simultaneously that are learned by the first layer separately. The next layer merges all input predictor sets, exploring common relations among them. In particular, we contrast the network regression fed by the connectivity indicators extracted within all three frequency rhythms against the widely used extracting approach from μ and $\beta_{\mathrm{low}}$.

Besides the *R*-squared value, the estimates of *MAE* and *RMSE* are also computed to evaluate the DRN performance, accounting for the influence of the adopted leave-one-out cross-validation strategy on the DRN performance. Concerning the examined graph indexes, Table 3 shows that the strength and clustering coefficient result in similar prediction performance. Also, their combination performs comparably to their separate training. In turn, the use of indicators directly based on phase synchronization allows producing comparative assessments with the graph indexes, meaning that the network regression can handle FC indicators with a lower complexity of computation.

Overall, the network regression reaches an *R*-squared value as high as 0.79 on average across the FC indicators extracted from DBI and 0.45 from DBII, respectively, outperforming all previous baseline linear regression outcomes displayed in Table 1. Moreover, the joint use of all frequency bands achieves better predictive performance than the two-rhythms ensemble in each EEG collection evaluated. Nevertheless, the prediction errors assessed by *MAE* and *RMSE* are still high. Even worse, the corresponding error values for DBI are lower than those estimated for DBII due to its greater variability.

Still, the DRN’s performance can be enhanced by evaluating its robustness against the noisy input sets, making the data-driven the data-driven regressor provide overfitting prediction assessments. To cope with this issue, we consider two strategies for improving the DRN robustness: Firstly, the thresholding method is incorporated, usually performed in functional connectivity analysis at the preprocessing stage, to remove false connections and noise. Following the procedure in [53], we fix the proportional thresholding rule to 0.7, preserving a sufficient amount of links under a value of *p* ≤ 0.1. Secondly, the leave-one-out cross-validation is further refined by incorporating the Monte Carlo dropout layers, containing neurons with a probability of being ignored during training and validation. Therefore, both assessments in Equation (8) and Equation (9) are recomputed by averaging over *Q* iterations of the Monte Carlo dropout applied to the dense DRN layers. It should be noted that the dropout rate is expected to be low due to the relatively small amount of input data in both databases tested. So, we fix the dropout rate heuristically to 0.2 while the number of iterations adjusts to Q=100.

Table 4 displays the regression performance computed by the leave-one-out cross-validation together with Monte Carlo dropout, revealing that the prediction improves notably because of the neurons with a probability of being ignored during training and validation. On average, the *R*-squared value reduces by 10% for DBI, but the prediction errors of *MAE* and *RMSE* fall by nearly half. In the case of DBII, the *R*-squared value rises by 20% while the prediction errors shrink by almost 40%. Once again, the extraction from three frequency rhythms is more effective. Additionally, the performance results using the thresholding procedure seem to improve the prediction assessment (for which the input predictor sets are denoted with *), but to some extent. As a result, the improved validation procedure combined with thresholding reduces the overfitting prediction effect, making it more effective in input measures with higher variability.

**Figure 4:**
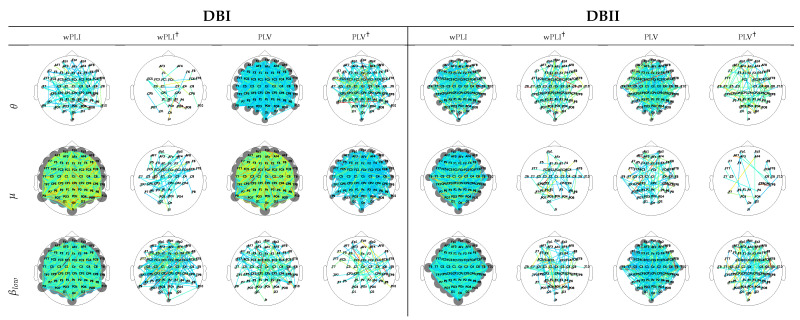
DNR weights mostly supporting the prediction performance, learned for *wPLI* and *PLV* predictors using two validation scenarios: leave-one-out cross-validation and leave-one-out cross-validation with Monte Carlo dropout (noted with †).

For interpretation purposes, Figure 4 depicts the DNR weights that mostly support the prediction performance, produced by *wPLI* and *PLV* after introducing the leave-one-out cross-validation. For DBI, the former measure weights (left column) are robust and spread all over the scalp, as happens with the latter FC measure (third column). DBII faces a similar situation (fifth and seventh columns), though providing fewer estimates. This result can be explained because of the higher complexity of DBII. Next, the use of leave-one-out cross-validation with Monte Carlo dropout (noted with ${}^{†}$) allows the number of contributing links to decrease sharply, therefore avoiding the DNR overfitting, as seen in all even columns regardless of the validated data collection. Note that in this case, all rhythms contribute, though to a different extent. Besides, most of the relevant links appear over the frontal-occipital and parietal-occipital areas directly related to the MI responses [54].

### 4.3. Enhanced DNR Prediction Assessment Using Subject Efficiency Clustering

To improve the regression performance, we determine the differences in neural responses among the categorized users depending on their motor skills as a critical factor affecting the data-driven estimator in Equation (6). Therefore, we find the number of subject groups from the MI classification performance using a clustering approach. The Silhouette score-based cost is utilized, finding three clusters and then applying the *k*-means algorithm to compute each subject membership.

**Figure 5:**
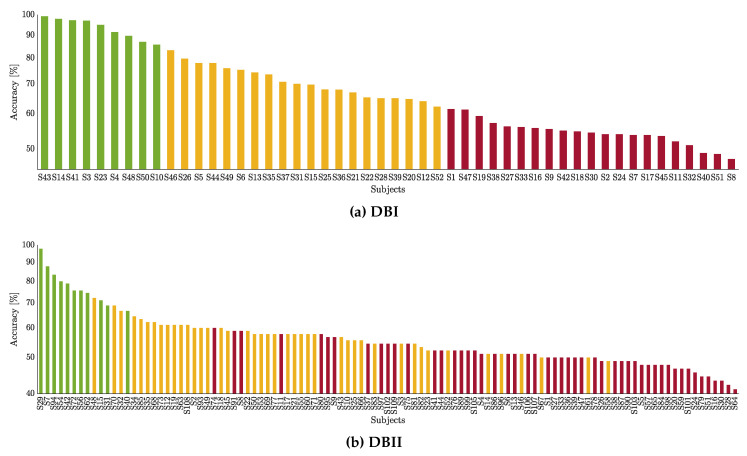
Partitions of individuals clustered by the CSP-based accuracy within the motor imagery interval. Each subject performance is painted according to his appraised inefficiency partition: Group I (green), Group II (yellow), and Group III (red).

The classification performance is presented in Figure 5 for the studied databases. A feature selection strategy is applied over the well-known CSP-based features to predict the MI label based on a Linear Discriminant Analysis classifier. A 10×10-fold cross-validation scheme is adopted. Thus, the three obtained groups are depicted in color bars: Group I contains the subjects performing the best (denoted by the green color), Group II with the subjects with intermediate accuracy (yellow color), Group III with the worst-performing subjects (red color). As seen, while DBI holds compactly distributed clusters, the neighboring groups are mixed in DBII. Of note, Group I includes the lowest number of individuals (10% in DBII), whereas groups II and III involve the remaining part.

For these cases, we calculate the DRN prediction with the performance improved. That is, we test the functional connectivity indicators of all three frequency rhythms extracted from resting-state data together with the leave-one-out cross-validation, including Monte Carlo dropout layers. Table 5 shows that the prediction analysis improves (the values of *R*-squared increase while the errors decrease) regardless of the subject group under consideration. Moreover, the improvement becomes higher for DBII, which means that the regression analysis using partitions can be also effective in databases with more complex EEG measurements.

The next aspect refers to the assessment of resting-state activation on a reduced number of electrodes. To this end, we evaluate the DRN performance for the predictor sets extracted over the sensorimotor area, selecting the following electrodes, as suggested in [44]: (FC1, FC2, FC3, FC4, FC5, FC6, Cz, C1, C2, C3, C4, C5, C6, CPz, CP1, CP2, CP3, CP4, CP5, and CP6). In Table 5, the lower part of each database presents the assessments computed for the sensorimotor zone (denoted with ^⋆^), showing that the channel selection strategy also improves the performance of every subject partition compared to the corresponding values in Table 4 obtained by the whole set of individuals. Nevertheless, the incorporation of the total number of electrodes increases in a higher degree the DNR prediction.

**Figure 6:**
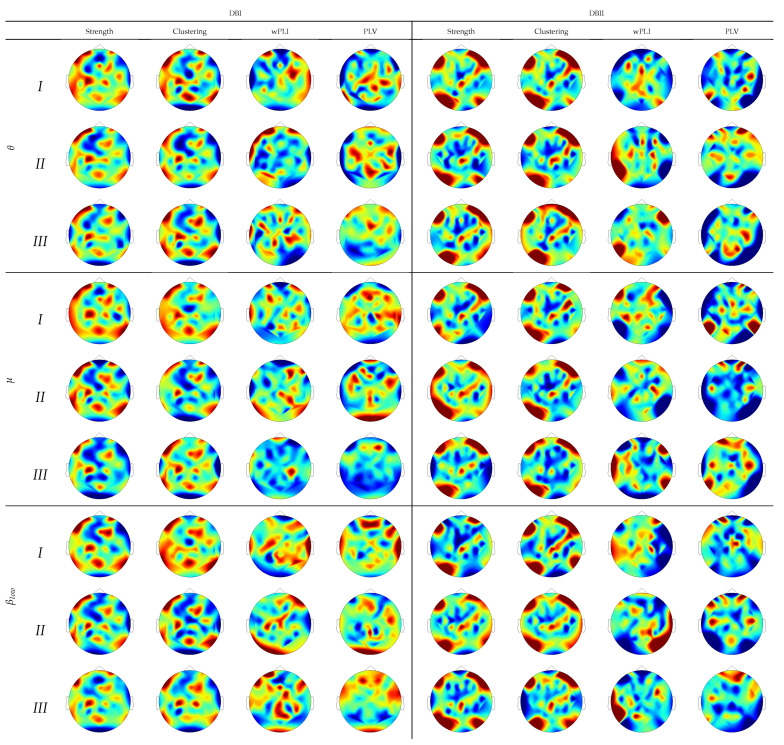
Topograms depicting the DNR weights performed by each inefficiency subject partition using FC predictors.

To provide further interpretation of the assessed regression weights, Figure 6 draws their estimates over the scalp surface performed by each subject partition. In DBI, the topograms of groups I and II are similar regardless of the extracted rhythm, especially for both graph indexes’ relevance values. Instead, Group-III subjects behave differently and tend to be smaller and more spread, as noticed on the topograms performed by either connectivity measure. Note that the FC measures emphasize the sensorimotor more than the graph indexes that highlight the occipital and frontal zones related to attention and visual tasks. Therefore, the figures performed by DBII are comparable to DBI but hold more variability.

### 4.4. Network Regression Prediction of Initial-Training Synchronization

To assess the initial training synchronization, we evaluate the DNR performance of predicting the MI accuracy at each run, which holds few trials and is affected by the learning changes. We evaluate two wakefulness data situations for extracting the FC predictor (namely, before the cue-onset of the conventional MI trial timing noted as $T_{0}$ and resting-state noted as $T_{rs}$). As the FC predictor, we only consider the *PLV* measure that, together with the single-run target accuracy vector, feeds the DNR estimator. This choice of FC is conditioned by its feasibility to be extracted in a single-trial mode from wakefulness data.

Table 6 presents the prediction capability of the synchronization behavior appraised by DNR in each subject partition. Compared to the all set performance, the FC extracted from resting-state data increasing each group’s prediction, meaning that the network can predict under a reduced number of trials per run to some extent. The alternative case of extracting *PLV* from $T_{0}$ enables a further improvement in each group’s prediction capability. It is worth noting that the *R*-squared value remains high over the run sequence, particularly in Group III with the subjects that need more guidance for promoting BCI skills. Overall, this test result of $T_{0}$ raises the possibility of the initial-training assessment carried out without additional EEG data acquisition. In the database DBII with considerable variability, however, the target response vector computed within each run yields a very low accuracy due to the lack of statistics (only 14 trials per run) so that the DRN performance drops noticeably, resulting in *R*-squared values below 0.2.

Utilizing a transfer-learning approach, we train the DNR estimator to cope with this issue, gathering the values learned from each run’s MI data with the weights inferred at the predecessor run. For DBI and DBII, Table 6 also displays the outcomes achieved by both EEG collections (extracting *PLV* from $T_{0}$) and shows that the DNR prediction ability outperforms in all three subject partitions the former strategies evaluated for predicting Initial-training Synchronization. As a result, each subject’s competence can be prognosticated with a high enough level after each run to carry out procedures, aiming at improving his performance in practicing MI tasks.

It is worth noting that the use of functional connectivity measures in BCI inefficiency prediction is still in the exploring stage rather than the power-based predictors extracted from the sensorimotor rhythms. Table 7 compares several works recently presented that employ correlates between accuracy and SMR or FC indicators, showing that the latter predictors combined with DNR are promising.

## 5. Concluding Remarks

Here, we develop a methodology for predicting MI practicing’s neurophysiological inefficiency using EEG phase synchronization measures. A deep network regression evaluates over 150 subjects’ predicting capability in assessing the pre-training desynchronization and the initial training synchronization. The prediction estimates should help determine whether a specific user needs to undergo an additional calibration, supplying interpretation of subjects’ learning properties. Although our algorithm training can be time-consuming, growing considerably as the database set increases, such a training stage can be implemented offline. Once the Deep Network Regressor’s weights are learned, our predictor evaluation is as fast as baseline models, enabling real-time applications like the run-based prediction of initial-training synchronization.

From the obtained results of validation, the following aspects are to be emphasized:

*Electrophysiological predictors based on functional connectivity*. We explore the Phase Locking Value and Weighted Phase Locking Index as connectivity measures together with their brain graph predictors (strength and clustering coefficient) to build a predictive regression model of BCI control. From the obtained results for the linear regression model (simple Table 1 and multiple Table 2), we can conclude that the FC predictors extracted from resting-state enable fair values of prediction performance (*R*-squared below 0.4) with notable variations, regardless of the input arrangement configuration employed. This behavior worsens in DBII that is an EEG collection with greater structure variableness.

With regard to DNR, all considered FC predictors present similar and even performance, reaching more competitive prediction values (see Table 3, Table 4 and Table 5). In terms of providing interpretation, the DNR weights mostly supporting the prediction performance are comparable in *wPLI* and *PLV* predictors (see Figure 4). One more consideration is the limited effectiveness of the thresholding method, usually performed to remove false connections and noise. The thresholding performance may be jeopardized by the high intrasubject variability, demanding the application of subject-related tuning algorithms. Therefore, the network regression models ease the need for elaborate feature extraction procedures based on functional connectivity analysis. It is worth noting that the network regression estimator benefits from all considered rhythms (i.e., $\theta +\mu +\beta_{\mathrm{low}}$), though each contributes differently.

*Quality of network regression models.* While widely-common procedures can appraise linear regression models’ statistical significance, assessing and enhancing network regression models’ prediction quality is a much more challenging task because of the risk of overfitting [56]. Here, we propose the leave-one-out cross-validation that includes Monte Carlo dropout layers (holding neurons with a probability of being ignored during training and validation) for decreasing the probability that the learned rules from specific training data cannot be generalized to new observations. As a result, the DNR prediction errors of *MAE* and *RMSE* fall by nearly half (see Table 4). Furthermore, including the Monte Carlo dropout layers allows selecting a reduced set of FC links enhancing the prediction performance (see Figure 4). Consequently, this aspect improves the physiological interpretability of network regression models.

In practice, assessment of resting-state activation is frequently performed with a reduced number of electrodes to reduce computational complexity and the set-up time. To this end, we evaluate the DRN performance for the predictor sets extracted over the sensorimotor area, showing that the channel selection strategy underperforms the whole electrode set’s inclusion. This issue becomes more manifest in subjects with a more prominent EEG variability (that is, high BCI inefficiency). As suggested in [57], the learned network weights depend on the variability resulting from the channel selection used, making the prediction performance vary notably from one subject partition to another.

*Regression assessments using subject clustering of BCI inefficiency.* One more issue impacting the regression prediction is the user’s categorization depending on their SMR activity and classifier performance during the MI runs. The obtained results show that the prediction performance improves (the values of *R*-squared increase while the errors decrease) regardless of the subject group under consideration. Therefore, we hypothesize that the regression analysis using partitions may be more effective in databases with complex EEG measurements. Consequently, clustering combined with DNR models enhances understanding of the factors influencing subjects’ accuracy performance with significant BCI inefficiency.

*DNR prediction with transfer learning.* We also assess the DNR performance of predicting the MI accuracy at each run using the single-trial *PLV* predictor of wakefulness data. However, we associate the values learned from each run’s MI data with the weights inferred at the predecessor run to deal with the few-trials sets. Thus, compared to the all set performance, the initial-training synchronization prediction increases in each group of individuals.

For future work, the authors plan to enhance FC predictors’ feature extraction, providing a better understanding of their impact and interaction on BCI-related tasks to identify potential non-learners.

Profiting from MI-based BCI learning progression, dynamic network regression models must be developed to capture the sequence regression’s latent trends. In this line of analysis, the cluster-based enhancing procedure and the vector accuracy response should also account for FC predictors’ dynamic behavior. Intending to improve the DNR prediction, an extended panel of standardized and validated psychological questionnaires are to be included within the network estimator, accounting for user’s specific characteristics like daily motor activity and age.

One more aspect to explore is to adjust the DNR pipeline to learn the weights for supporting prediction in a broader clinical application class, relying on the ability of deep learning architectures to extract complex random structures from EEG data.

## 6. Author Resume

- **Julian Caicedo-Acosta** received his undergraduate degree in electronic engineering (2018) and his M.Sc. degree in engineering industrial automation (2019) from the Universidad Nacional de Colombia. Currently, he is a PhD student at the same university. His research interests include machine learning, signal processing and bioengineering.
- **German A. Castaño** received his undergraduate degree in economics (1986) and business administration (1987), and his specialization of informatics administration (1993) from the Universidad Nacional de Colombia. Currently, he is a Professor in the Department of Administration at the Universidad Nacional de Colombia – Manizales. In addition, he is Chairman of the “Grupo de investigación Cultura de la Calidad en la Educación”at the same university. His research interests include quality of education, peace, and post-conflict.
- **Carlos Acosta-Medina** received a B.S. degree in Mathematics from Universidad de Sucre in Colombia in 1996. In 2000, he received a M.Sc. degree in mathematics, and in 2008 a Ph.D. in mathematics, both of them from Universidad Nacional de Colombia—Sede Medellín. Currently he is Associated Professor at Universidad Nacional de Colombia—Sede Manizales. His research interests are Regularization, Conservation Laws, and Discrete Mollification.
- **Andres Alvarez-Meza** received his undergraduate degree in electronic engineering (2009), his M.Sc. degree in engineering industrial automation (2011), and his Ph.D. in engineering—automatics (2015) from the Universidad Nacional de Colombia. Currently, he is a Professor in the Department of Electrical, Electronic, and Computation Engineering at the Universidad Nacional de Colombia – Manizales. His research interests include machine learning and signal processing.
- **German Castellanos-Dominguez** received his undergraduate degree in radiotechnical systems and his Ph.D. in processing devices and systems from the Moscow Technical University of communications and Informatics, in 1985 and 1990 respectively. Currently, he is a Professor in the Department of Electrical, Electronic, and Computation Engineering at the Universidad Nacional de Colombia, Manizales. In addition, he is Chairman of the GCPDS at the same university. His teaching and research interests include information and signal theory, digital signal processing, and bioengineering.

## Figures and Tables

**Table 1:** Predicting performance of the FC predictors extracted from the resting-state data, employing the matrix indicator, the average, and the best index computed across the whole channel set. Notation *mean* stands for the indicator $R^{2}$ averaged across the frequency bands. Abbreviation *na* is not applicable.

	DBI						DBII					
Ω	*Strenght*											
[*Hz*]	*multichannel*		*average*		*best channel*		*multichannel*		*average*		*best channel*	
	$R^{2}$	*p*-val	$R^{2}$	*p-*val	$R^{2}$	*p-*val	$R^{2}$	*p*-val	$R^{2}$	*p-*val	$R^{2}$	*p-*val
θ	0.472	>0.01	0.088	0.542	0.322	0.022	0.090	0.360	0.098	0.318	0.223	0.022
μ	0.296	0.036	0.268	0.059	0.382	0.006	0.155	0.113	0.169	0.083	0.355	0.002
$\beta_{\mathrm{low}}$	0.069	0.633	0.131	0.361	0.348	0.013	0.032	0.742	0.124	0.206	0.153	0.118
*mean*	*0.279*		*0.162*		*0.351*		*0.092*		*0.130*		*0.243*	
	*Clustering coefficient*											
θ	0.180	0.210	0.177	0.217	0.430	0.001	0.205	0.035	0.055	0.573	0.231	0.017
μ	0.178	0.215	0.295	0.037	0.363	0.009	0.139	0.154	0.150	0.124	0.387	>0.01
$\beta_{\mathrm{low}}$	0.167	0.316	0.307	0.029	0.255	0.073	0.225	0.020	0.143	0.145	0.164	0.092
*mean*	*0.167*		*0.260*		*0.349*		*0.190*		*0.117*		*0.261*	
	*wPLI*											
θ	0.104	0.468	0.028	0.845	na	na	0.248	0.010	0.300	0.001	na	na
μ	0.418	0.002	0.352	0.012	na	na	0.075	0.444	0.482	>0.01	na	na
$\beta_{\mathrm{low}}$	0.269	0.058	0.203	0.155	na	na	0.202	0.037	0.346	>0.01	na	na
*mean*	*0.263*		*0.194*				*0.175*		*0.376*			
	*PLV*											
θ	0.471	>0.01	0.406	0.003	na	na	0.086	0.378	0.088	0.368	na	na
μ	0.454	0.001	0.627	0	na	na	0.066	0.497	0.261	0.006	na	na
$\beta_{\mathrm{low}}$	0.425	0.002	0.305	0.030	na	na	0.064	0.514	0.317	0.001	na	na
*mean*	*0.450*		*0.445*				*0.074*		*0.222*			

**Table 2:** Prediction performance of the multiple linear regression for the graph indexes extracted from the resting-state data.

	DBI				DBII			
Ω		*Average*		*Multichannel*		*Average*		*Multichannel*	
	$R^{2}$	***p*** **-val**	$R^{2}$	***p*** **-val**	$R^{2}$	***p*** **-val**	$R^{2}$	***p*** **-val**
θ	0.192	0.180	0.312	0.026	0.234	0.102	0.085	0.387
μ	0.134	0.351	0.173	0.228	0.213	0.137	0.121	0.222
$\beta_{\mathrm{low}}$	0.032	0.822	0.008	0.951	0.101	0.484	0.116	0.239
*mean*	*0.120*		*0.164*		*0.183*		*0.107*	

**Table 3:** Obtained prediction performance of the *Wide&Deep* neural network regression fed by the tested functional connectivity predictors extracted from the resting-state data, contrasting two different wide path configurations of rhythm extraction: considered rh and $\mu +\beta_{\mathrm{low}}$.

$\theta +\mu +\beta_{\mathrm{low}}$						
Ω	**DBI**			**DBII**		
	$R^{2}$	***RMSE***	***MAE***	$R^{2}$	***RMSE***	***MAE***
Strength	0.787	0.488	0.424	0.412	0.440	0.393
Clus. Coefficient	**0.804**	0.444	0.367	0.472	0.471	0.436
Both indexes	0.800	0.419	0.353	0.321	0.367	0.314
*wPLI*	0.791	0.476	0.396	**0.591**	0.448	0.391
*PLV*	0.785	0.468	0.395	0.465	0.421	0.374
*mean*	*0.793*	*0.454*	*0.386*	*0.452*	*0.390*	*0.343*
$\mu +\beta_{\mathrm{low}}$						
Strength	0.766	0.470	0.388	**0.525**	0.406	0.342
Clus. Coefficient	0.695	0.475	0.396	0.456	0.372	0.316
Both indexes	**0.788**	0.464	0.382	0.392	0.404	0.338
*wPLI*	0.772	0.544	0.480	**0.525**	0.354	0.305
*PLV*	0.700	0.426	0.353	0.362	0.405	0.350
*mean*	*0.744*	*0.485*	*0.410*	*0.429*	*0.367*	*0.309*

**Table 4:** Prediction performance of the *Wide&Deep* neural network regression fed by the tested functional connectivity indicators extracted from the resting-state data, employing the validation procedure that includes the Monte Carlo dropout layers combined with the threshold procedure. The input predictor sets denoted with * are thresholded. Bold numbers show the best result of each experiment.

$\theta +\mu +\beta_{\mathrm{low}}$						
Ω	**DBI**			**DBII**		
	$R^{2}$	***RMSE***	***MAE***	$R^{2}$	***RMSE***	***MAE***
Strength	0.731	0.247	0.212	0.341	0.293	0.247
Strength *	0.763	0.184	0.142	0.646	0.216	0.179
Clus. Coefficient	0.453	0.238	0.192	0.635	0.240	0.201
Clus. Coefficient *	0.753	0.208	0.174	0.698	0.242	0.202
Both indexes	**0.810**	0.145	0.110	**0.741**	0.165	0.127
Both indexes *	0.672	0.186	0.145	0.701	0.167	0.132
*PLV*	0.730	0.183	0.140	0.519	0.391	0.355
*wPLI*	0.795	0.146	0.112	0.573	0.216	0.173
*mean*	*0.713*	*0.192*	*0.153*	*0.606*	*0.241*	*0.202*
$\mu +\beta_{\mathrm{low}}$						
Strength	0.666	0.200	0.151	0.270	0.252	0.198
Strength *	0.781	0.168	0.128	0.439	0.277	0.239
Clus. Coefficient	0.795	0.155	0.126	**0.816**	0.202	0.177
Clus. Coefficient *	0.676	0.186	0.155	0.771	0.180	0.150
Both indexes	0.597	0.208	0.158	0.629	0.232	0.187
Both indexes *	0.666	0.182	0.150	0.693	0.207	0.170
*PLV*	0.742	0.210	0.173	0.344	0.272	0.213
*wPLI*	**0.864**	0.123	0.098	0.763	0.212	0.185
*mean*	*0.660*	*0.226*	*0.153*	*0.547*	*0.229*	*0.189*

**Table 5:** DNR Performance of the functional connectivity (FC) predictors derived from the resting-state data achieved by each subject partition. Prediction is carried employing Monte Carlo dropout and the wide path configuration of all extracted rhythms ($\theta +\mu +\beta_{\mathrm{low}}$).

DBI									
Ω	***I*** **(8)**			***II*** **(15)**			***III*** **(27)**		
	$R^{2}$	***RMSE***	***MAE***	$R^{2}$	***RMSE***	***MAE***	$R^{2}$	***RMSE***	***MAE***
Strength	0.625	0.311	0.255	0.868	0.140	0.111	0.799	0.152	0.115
Clus. Coefficient	0.792	0.260	0.170	0.853	0.160	0.105	0.777	0.179	0.137
Both indexes	0.828	0.248	0.198	0.767	0.229	0.166	0.750	0.169	0.134
*wPLI*	0.872	0.228	0.190	0.907	0.141	0.113	0.958	0.073	0.061
*PLV*	0.947	0.164	0.124	0.883	0.140	0.112	0.939	0.095	0.072
*mean*	0.813	0.242	0.187	0.856	0.162	0.121	0.845	0.134	0.104
*Gain* [%]	*+14*	*+74*	*78*	*+20*	*−16*	*−21*	*+18*	*−30*	*−32*
Strength ^⋆^	0.860	0.210	0.191	0.636	0.265	0.166	0.788	0.161	0.136
Clus. Coefficient ^⋆^	0.973	0.109	0.075	0.690	0.214	0.160	0.798	0.142	0.108
Both indexes ^⋆^	0.946	0.192	0.147	0.819	0.171	0.115	0.671	0.190	0.146
*wPLI* ^⋆^	0.570	0.317	0.241	0.921	0.109	0.080	0.830	0.152	0.119
*PLV* ^⋆^	0.841	0.189	0.103	0.802	0.185	0.141	0.739	0.167	0.135
*mean*	0.838	0.203	0.151	0.773	0.188	0.132	0.765	0.162	0.128
*Gain* [%] ^⋆^	+17	+6	−1	+8	−2	−14	+7	−15	−16
**DBII**									
	*I* (11)			*II* (43)			*III* (51)		
Strength	0.878	0.139	0.119	0.723	0.197	0.163	0.837	0.193	0.157
Clus. Coefficient	0.940	0.089	0.075	0.618	0.213	0.173	0.885	0.128	0.099
Both indexes	0.884	0.127	0.090	0.499	0.297	0.240	0.846	0.157	0.13
*wPLI*	0.971	0.066	0.039	0.716	0.185	0.150	0.749	0.142	0.110
*PLV*	0.824	0.166	0.118	0.804	0.161	0.137	0.833	0.130	0.103
*mean*	0.899	0.117	0.088	0.672	0.210	0.172	0.830	0.150	0.119
*Gain* [%]	+48	−51	−56	+11	−13	−15	+37	−38	−41
Strength ^⋆^	0.806	0.164	0.105	0.626	0.288	0.250	0.715	0.268	0.231
Clus. Coefficient ^⋆^	0.803	0.183	0.147	0.713	0.224	0.184	0.673	0.673	0.308
Both indexes ^⋆^	0.889	0.159	0.113	0.130	0.286	0.229	0.631	0.221	0.167
*wPLI* ^⋆^	0.618	0.286	0.238	0.656	0.198	0.154	0.638	0.210	0.173
*PLV* ^⋆^	0.882	0.128	0.092	0.740	0.132	0.100	0.825	0.155	0.125
*mean* ^⋆^	0.799	0.184	0.139	0.573	0.225	0.183	0.696	0.305	0.200
*Gain* [%] ^⋆^	+32	−24	−31	−5	−7	−9	+15	+26	−1

**Table 6:** DNR performance in predicting initial-training Synchronization, employing Monte Carlo dropout and the wide path configuration of all extracted rhythms ($\theta +\mu +\beta_{\mathrm{low}}$). The network regression is fed by the *PLV* predictor as the only single-trial FC indicator.

DBI												
**Run**	***I*** **(8)**			***II*** **(15)**			***III*** **(27)**			**All Set**		
	$R^{2}$	***RMSE***	***MAE***	$R^{2}$	***RMSE***	***MAE***	$R^{2}$	***RMSE***	***MAE***	$R^{2}$	***RMSE***	***MAE***
	Resting-State vs. Single-Run Accuracy Target											
1	0.601	0.245	0.192	0.627	0.214	0.143	0.888	0.126	0.085	0.814	0.192	0.158
2	0.897	0.200	0.158	0.745	0.214	0.176	0.874	0.117	0.087	0.795	0.141	0.109
3	0.862	0.184	0.121	0.655	0.275	0.146	0.910	0.123	0.099	0.756	0.226	0.226
4	0.917	0.172	0.123	0.972	0.069	0.053	0.936	0.110	0.090	0.890	0.109	0.089
5	0.550	0.293	0.217	0.906	0.131	0.106	0.819	0.171	0.130	0.689	0.231	0.190
*mean*	*0.765*	*0.219*	*0.162*	*0.781*	*0.181*	*0.125*	*0.885*	*0.129*	*0.098*	*0.789*	*0.180*	*0.154*
	single-run before-unset interval vs. single-run accuracy target											
1	0.733	0.318	0.280	0.751	0.171	0.110	0.914	0.110	0.080	0.809	0.134	0.104
2	0.966	0.113	0.074	0.782	0.187	0.116	0.777	0.209	0.162	0.791	0.140	0.107
3	0.900	0.189	0.129	0.678	0.248	0.135	0.855	0.167	0.123	0.806	0.168	0.126
4	0.924	0.134	0.100	0.943	0.120	0.093	0.893	0.102	0.083	0.847	0.131	0.110
5	0.678	0.265	0.233	0.911	0.130	0.102	0.741	0.192	0.137	0.835	0.165	0.136
*mean*	*0.840*	*0.204*	*0.163*	*0.813*	*0.171*	*0.111*	*0.836*	*0.156*	*0.117*	*0.818*	***0.148***	*0.117*
	single-run before-unset interval vs. all-run accuracy target using transfer learning											
1	0.875	0.187	0.142	0.864	0.144	0.109	0.956	0.077	0.058	0.848	0.129	0.101
2	0.954	0.143	0.102	0.918	0.122	0.093	0.947	0.102	0.081	0.813	0.150	0.127
3	0.965	0.137	0.094	0.943	0.105	0.086	0.924	0.089	0.067	0.758	0.156	0.110
4	0.983	0.107	0.089	0.932	0.109	0.089	0.941	0.088	0.065	0.786	0.192	0.160
5	0.981	0.105	0.085	0.952	0.095	0.070	0.942	0.086	0.059	0.733	0.248	0.210
*mean*	***0.952***	***0.136***	***0.102***	***0.922***	***0.115***	***0.089***	***0.942***	***0.085***	***0.066***	***0.788***	***0.175***	***0.142***
	**DBII**											
	single-run before-unset interval vs all-run accuracy target using transfer learning											
1	0.746	0.201	0.127	0.629	0.185	0.152	0.708	0.175	0.138	0.340	0.236	0.190
2	0.792	0.184	0.150	0.630	0.220	0.173	0.596	0.225	0.176	0.482	0.282	0.233
3	0.877	0.146	0.115	0.755	0.168	0.134	0.577	0.225	0.183	0.441	0.269	0.231
*mean*	0.805	0.177	0.131	0.671	0.191	0.153	0.627	0.208	0.166	0.421	0.262	0.218

**Table 7:** Performance comparison with works recently presented that employ correlates between accuracy and sensorimotor rhythms (SMR) or FC indicators. S denotes the number of subjects in respective datasets.

SMR [41]		FC [55]		ERSP [38]			FC [44]			DRN DBI			DRN DBII		
$R^{2}$	sub	$R^{2}$	sub	$R^{2}$ LI	$R^{2}$ CAS	sub	$R^{2}$	$R^{2}$ single-run	sub	$R^{2}$	$R^{2}$ single-run	sub	$R^{2}$	$R^{2}$ single-run	sub
0.37	80	0.53	80	-0.73	0.64	34	0.31	0.54	54	0.86	0.84	50	0.81	0.48	104

## Data Availability

The databases used in this study are public and can be found at the following links: **DBI:** http://gigadb.org/dataset/100295, accessde on 30 January 2021; **DBII:** https://physionet.org/content/eegmmidb/1.0.0/, accessde on 30 January 2021.
